# Comparison of excess post-exercise oxygen consumption of different exercises in normal weight obesity women

**DOI:** 10.20463/jenb.2019.0013

**Published:** 2019-06-30

**Authors:** Won-Sang Jung, Hyejung Hwang, Jisu Kim, Hun-Young Park, Kiwon Lim

**Affiliations:** 1 Physical Activity and Performance Institute (PAPI), Konkuk University, Seoul Republic of Korea; 2 Department of Physical Education, Konkuk University, Seoul Republic of Korea

**Keywords:** Continuous exercise, Interval exercise, Accumulation of short duration exercise, excess post-exercise oxygen consumption (EPOC), normal weight obesity (NWO) women

## Abstract

**[Purpose]:**

The purpose of this study was to compare the excess post-exercise oxygen consumption (EPOC) between different types of exercises in women with normal weight obesity (NWO).

**[Methods]:**

Nine university students with NWO having body mass index <25 kg/m^2^ and body fat percentage >30% participated in the study. First, continuous exercise (CEx) on an ergometer for 30 minutes at 60% of maximal oxygen consumption (VO_2max_) and interval exercise (IEx) at 80% VO_2max_ for 2 minutes were performed. This was followed by exercise performed at 40% VO_2max_ for 1 minute and at 80% VO_2max_ for 3 minutes, performed 6 times repeatedly for a total of 26 minutes. The accumulation of short duration exercise (AEx) was performed for 3-bouts of 10 minutes each at 60% VO_2max_.

**[Results]:**

The major findings were as follows: energy consumption during the exercises showed no significant difference between CEx, IEx, and AEx; EPOC was higher in IEx and AEx as compared to CEx for all dependent variables (e.g. total oxygen consumption, total calorie, summation of heart rate, and EPOC duration); and the lipid profile showed no significant difference.

**[Conclusion]:**

Our study confirmed that when homogenizing the energy expenditure for various exercises in NWO individuals, EPOC was higher in IEx and AEx than in CEx. Therefore, IEx and AEx can be considered as effective exercise methods for increasing energy expenditure in NWO females.

## INTRODUCTION

Recently, a new syndrome called normal weight obesity (NWO) among young Korean women has attracted attention. Based on the 2009-2010 National Health and Nutrition Examination Survey, the prevalence of NWO among adult women (aged >20 years) with normal body mass index (BMI) is about 30%, affecting every 1 out of 3 normal weight women^1^.

NWO is a combination of normal weight (BMI 18.5-24.9 kg/m^2^) with increased body fat percentage (BF% >30%), and is often seen in women in their twenties^2,3^. This can be attributed to the prevalence of wrong diets among these women, who focus only on external appearances and weight loss by cutting down on food intake without exercise^1,2^. NWO is not a physically apparent obesity because of the normal range BMI, but the decreased muscle mass and increased visceral fat is noticeable^4,5^. Visceral fat is accumulation of fat cells in the abdominal cavity, and it causes chronic inflammation as an endocrine organ secreting infectious cytokines^6^ that increases the incidence of various metabolic diseases risk ratio such as type 2 diabetes, hypertension, and hyperlipidemia^7^. The decrease in muscle mass is being magnified as an important health problem, because it causes insulin resistance, cardiovascular diseases, and increase in incidence of lifestyle-related diseases^5,8^. Since NWO in women in their twenties could be susceptible to various metabolic and cardiovascular diseases due to decreased muscle mass and increased visceral fat, active efforts should be made to prevent and improve metabolic diseases and cardiovascular diseases by increasing muscle mass and decreasing fat.

The American Academy of Sports Medicine recommends increasing physical activity or exercise to maintain health and promote fat loss, because regular exercise prevents weight gain and reduces the risk of obesity-related illnesses^9^. It was reported that resistance exercises and aerobic exercises were most effective in increasing muscle mass and decreasing fat to prevent or improve obesity^10,11^. Resistance exercise has been traditionally effective in increasing muscle mass and strength, and recommended for the prevention and treatment of musculoskeletal disorders, hypertension, and various metabolic and cardiovascular diseases^12^. Furthermore, it has been emphasized that resistance exercise is essential for improving body composition and physical fitness in women with relatively low muscle mass and strength^9^. Previous studies have also reported that it is effective in NWO women^13^. 

Continuous aerobic exercise (CEx) is an effective method to improve cardiopulmonary capacity and reduce body fat mass. It also contributes in reduction of visceral fat and increases the ability to control blood glucose, thus slowing the type 2 diabetes progress and reducing blood pressure, total cholesterol (TC), low-density lipoprotein cholesterol (LDL-C), and triglycerides (TG)^10,14,15^. Moreover, its influence on alleviating symptoms related to quality of life and depression has been reported^16^. However, despite these advantages, CEx needs to be performed at least 20-60 minutes a day at moderate or high intensity at least 3 times a week, and it needs to be performed for 60-90 minutes a day to achieve significant weight loss of over 2,000 kcal per week^9,11,17^. It should be performed at high-intensity for >60 minutes for this health benefit, but only limited persistent aerobic exercise can be prescribed in people who cannot participate in prolonged exercise due to NWO or low fitness.

Interval exercise (IEx), which carries out high- and low-intensity exercises repeatedly, and accumulation of short duration exercise (AEx), a method of dividing the total amount of exercise per day into several smaller time intervals, have recently drawn attention^18,19^. It is reported that IEx increased the energy consumption during and after exercise, and has been effective in reducing body fat, improving insulin resistance, and lowering cardiovascular disease risk^20^. Moreover, AEx has similar health benefits as CEx with respect to energy consumption after exercise^19^. Nevertheless, there is still no clear conclusion, with some studies showing a positive effect on energy consumption during exercise and some showing no significant change^21,22^. Most studies have been conducted on both normal and obese women, but there has been no study on NWO women.

Therefore, the purpose of this study was to compare the excess post-exercise oxygen consumption (EPOC) during different exercises that spent the same amount of calories in NWO women, and to provide important basic data in planning exercise programs by presenting proper exercise methods for preventing NWO.

## METHODS

Nine university students (mean age 22.78 ± 1.56 years) with NWO, who did not train regularly in exercise, volunteered to participate in this study. NWO was defined as BMI <25 kg/m^2^ and %BF >30% for women^2,3^. G*Power 3.0 software^23^ was used to estimate the sample size, and the effect size was calculated as per the study by Greer et al.^24^. The effect size (a = 0.05, power 0.80, effect size 2.82) was calculated for 6 individuals. The exclusion criteria were unstable angina, recently cardiac infarction (4 weeks), uncompensated heart failure, severe valvular illness, pulmonary disease, uncontrolled hypertension, kidney failure, orthopedic/neurological limitations, cardiomyopathy, planned surgery during the research period, reluctance to sign the consent from, drug or alcohol abuse, or involvement in another study. All participants were fully acquainted with the nature of the study and informed of the experimental risks before signing a written consent form to participate. It was explicitly stated to the participants that they could withdraw from the study at any point. The pre-test research was explained and voluntary consent was obtained. All procedures of the study were approved by the Institutional Review Board of Konkuk University (7001355-201903-HR-305) in Korea and conducted according to the Declaration of Helsinki guidelines. The physical characteristics of the participants are shown in Table 1.

**Table 1.** Characteristics of the participants

Variables	Normal weight obesity (n=9)
Age (years)	22.78±1.56
Height (cm)	160.20±4.94
Weight (kg)	55.18±4.74
BMI (kg/m^2^)	21.49±1.26
Lean body mass (kg)	37.04±3.55
Fat mass (kg)	18.13±1.87
% fat mss (%)	32.91±2.23
VO_2max_	31.07±3.69

Data represented as mean ± standard deviation (BMI = body mass index; VO_2max_ = maximal oxygen consumption)

### Experimental design

To test the EPOC during and after CEx, IEx, and AEx, we used a balanced repeated measures crossover design. This approach entailed gathering data of the completed 3 training sessions by the participants on separate test days in a randomized order.

Each participant visited the laboratory 4 times. On the first visit, body composition test (InBody 770, Biospace Ltd, Seoul, Korea) and maximal cardiopulmonary exercise test (Quark CPET, Cosmed, Italy) were performed. Seventy-two hours after the maximal exercise on the first visit, CEx cycling (1 × 30 min)^9^, AEx cycling (3 × 10 min) at 60% of maximal oxygen consumption (VO_2max_)^19^, and IEx cycling at 40% or 80% of VO_2max_ were performed on the second, third, and fourth visits, respectively^18^. As soon as the exercise ended, participants came down from the bicycle, sat on a chair, and measured EPOC for 60 minutes.

### Pre-testing measurements

The participants performed maximal aerobic exercise test using a cycle ergometer (Aerobike, Combi 75 XL, Tokyo, Japan) in order to determine VO_2max_. The work rate was 50 rpm at 25 W for the first 2 minutes and was increased by 12.5 W every 2 minutes until exhaustion or until participants were unable to maintain 50 rpm. The criteria for reaching the true VO_2max_ was when a plateau in VO_2_ was reached despite increased intensity of exercise and a respiratory exchange ratio (RER) >1.15. The heart rate (HR) was measured using Polar 800 (Polar Electro, Kempele, Finland).

### Exercise training protocol

The participants visited the laboratory at 8 am following a 12-hour fasting and 48 hours of no vigorous physical activities, and consumed a standardized breakfast (2 loaves of bread [200 kcal], a boiled egg [80 kcal], orange juice [120 kcal]) 1 cup, and 1 cup water). Ambient room temperature was maintained at 23 ± 1℃. After 10 minutes in habituation period of quiet sitting, VO_2max_, ventilation, and RER were measured for 5 minutes with the average used as the baseline (BASE). 

**Figure 1.**
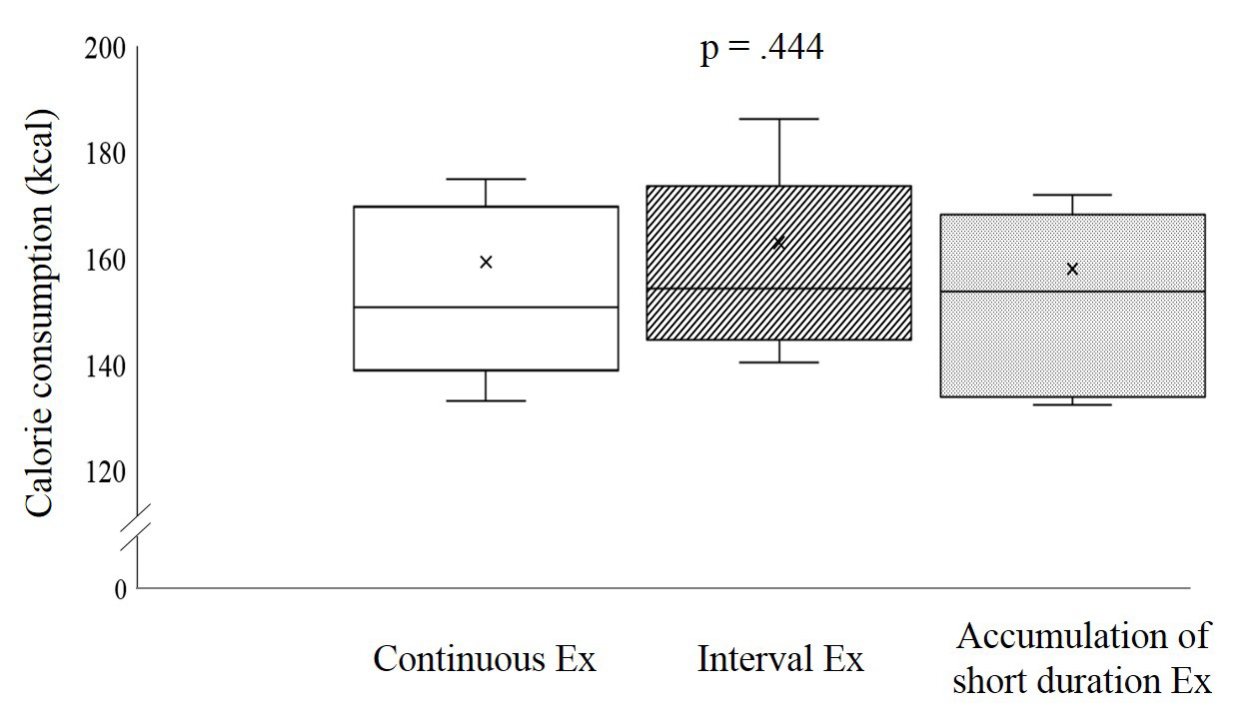
Comparison of calorie consumption during exercise

The CEx and IEx were performed on an ergometer at 60% of VO_2max_ for 30 minutes and at 80% of VO_2max_ for 2 minutes, respectively, during the first time. This was followed by exercise at 40% VO_2max_ for 1 minute and at 80% VO_2max_ for 3 minutes, performed 6 times repeatedly for a total of 26 minutes. AEx was performed for 30 minutes on the ergometer, 3 times with intensity of 60% VO_2max_, for 10-minute sessions separated by intervals of 1 hour. The same amount of calories were spent between the different exercises (Figure 1).

### EPOC measurement

Immediately after the exercise, participants were seated in a chair while relative VO_2_, absolute VO_2_, kcal, HR, and duration were monitored continuously for the first 60 minutes of recovery. Criterion for EPOC values determination was set at the time when VO_2_, HR, and RER values returned to the resting BASE.

Collection and analysis of lipid samples were performed before exercise, immediately after exercise, 30 minutes after exercise, and 60 minutes after exercise. The TC, TG, high-density lipoprotein cholesterol (HDL-C), and LDL-C values were measured using a portable digital lipid analyzer (SD LipidoCare, SD Biosensor, Inc., Seoul, Korea).

### Statistics

All statistical analyses were completed using IBM SPSS Statistics version 22 (SPSS Inc., Chicago, IL, USA). Data were presented as mean ± standard deviation. Normality of distribution of all outcome variables was verified using Kolmogorov-Smirnov test. One-way analysis of variance was used to compare the differences between the 3 protocols. The effects of condition on EPOC were analyzed using the mixed procedure. If main effects were statistically significant, post-hoc Bonferroni correction were performed. Effect sizes were calculated using partial Eta square (η^2^). All statistically significant values were set at p < 0.05.

## RESULTS

This study showed that the calorie expenditure during exercise was not significantly different between CEx, IEx, and AEx (p = .444, see Figure 1).

The results of the EPOC in Table 2 show that EPOC duration was longer in IEx and AEx as compared to CEx (25.22 ± 15.06 vs. 42.44 ± 14.06 vs. 45.00 ± 14.31, respectively, p < .01). The EPOC results were higher for IEx and AEx as compared to CEx for all variables: total VO2 (8028.42 ± 4856.21 vs. 13252.18 ± 4546.30 vs. 18037.22 ± 8970.81, respectively, p < .05), total VO2/kg (145.36 ± 87.53 vs. 238.19 ± 71.09 vs. 326.57 ± 164.30, respectively, p < .05), total Kcal (38.81 ± 23.06 vs. 63.54 ± 21.39 vs. 88.57 ± 43.03, respectively, p < .01), and summation of HR (2451.27 ± 1688.98 vs. 4435 ± 1688.76 vs. 5076.33± 2484.06, respectively, p < .05).

**Table 2.** Comparison of excess post-exercise oxygen consumption (EPOC) for different exercise types

Variables	Con Ex	Inter Ex	Accumul Ex	*η*^2^ (p) value
VO_2__total (mL/min)	8028.42±4856.21^a^	13252.18±4546.30^b^	18037.22±8970.81^b^	.435 (.010)*
VO_2_/kg_total (mL/min/kg)	145.36±87.53^a^	238.19±71.09^b^	326.57±164.30^b^	.434 (.011)*
Kcal_total (kcal/min)	38.81±23.06^a^	63.54±21.39^b^	88.57±43.03^b^	.460 (.007)**
HR_sum	2451.27±1688.98^a^	4435.81±1688.76^b^	5076.33±2484.06^b^	.423 (.012)*
EPOC duration (min)	25.22±15.06^a^	42.44±14.06^b^	45.00±14.31^b^	.515 (.003)**

Data represented as mean ± standard deviation

(CEx = continuous exercises, IEx = Interval exercise, AEx = Accumulation of short duration exercise,

VO_2_ = oxygen consumption, HR = heart rate, Sum = summation)

* p < .05, ** p < .01 Significant

a, b, c = Different alphabets indicate significant difference

**Figure 2.**
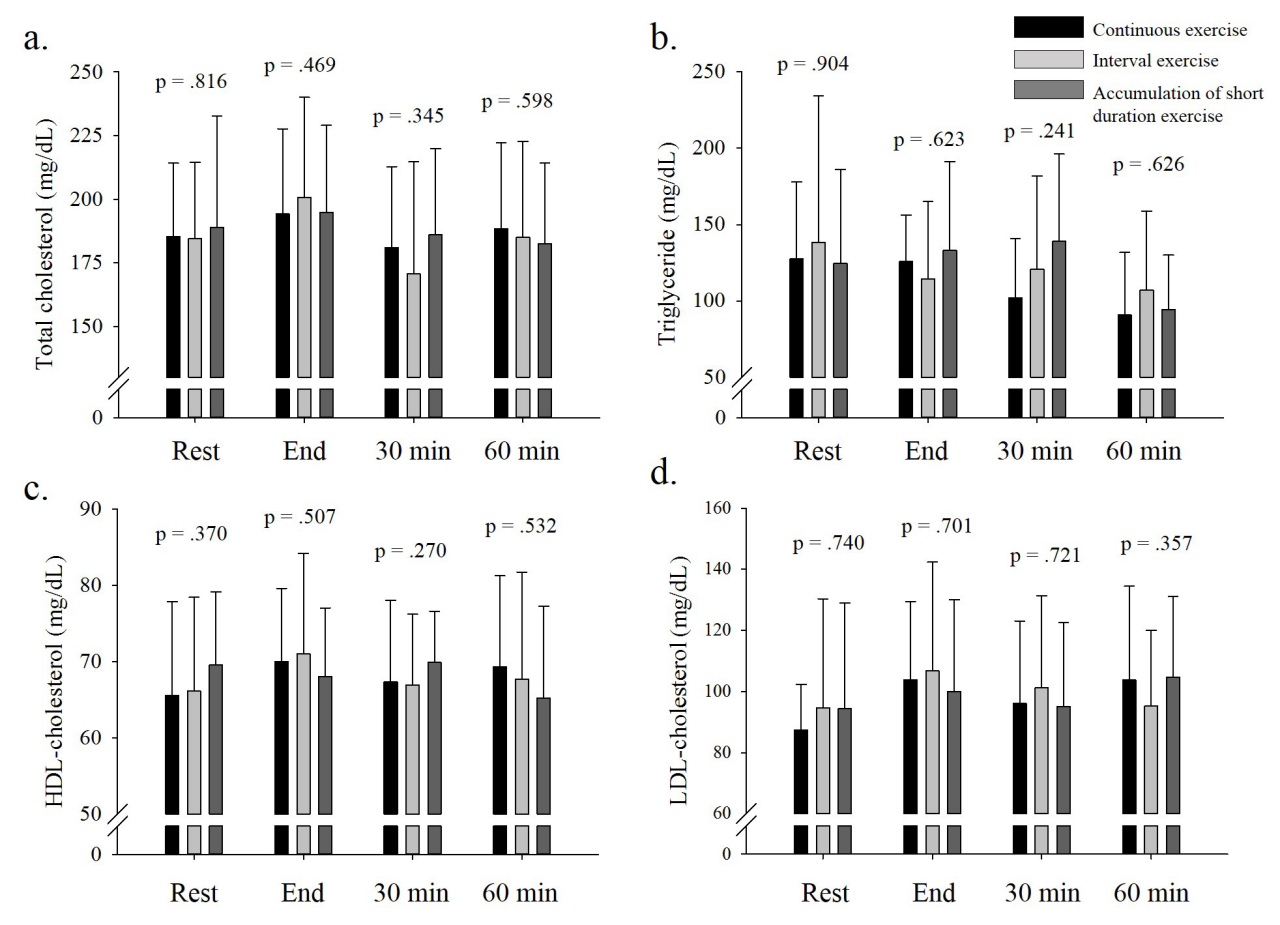
Comparison of lipid profile on EPOC in CEx vs IEx vs AEx

Comparison of lipid profile for EPOC in CEx, IEx, and AEx showed no significant difference in TC, TG, HDL-C, and LDL- C variables (p > .05, see Figure 2).

## DISCUSSION

Our study aimed to compare and analyze the EPOC for different exercises that spent the same amount of calories, and to suggest an appropriate exercise method to improve and prevent NWO. The main result of our study was that when energy consumption during the exercises was homogenized, IEx and AEx showed greater post-exercise energy expenditure than that of CEx.

In a previous study, Townsend et al.^25^ compared EPOC by performing CEx and IEx in university students, and reported a significant increase in EPOC of IEx as compared to CEx. Similarly, the study by Larsen et al.^26^ compared CEx and IEx results in adult male participants and reported a significant increase in EPOC of IEx as compared to CEx. The body needs energy during exercise; as exercise intensity and exercise duration increase, mitochondrial activity and oxygen consumption increases to produce more adenosine triphosphate (ATP)^27^. Additionally, during high-intensity exercise, the production of ATP depends on anoxic processes, and the oxygen deficiency occurring at the beginning of exercise is compensated by consuming more oxygen in the recovery period after exercise^28,29^. The reason for the increase in oxygen consumption after exercise is that the first depleted ATP-PCr is re-synthesized, oxygen is replenished in the intramuscular myoglobin, and it is consumed to supply dissolved oxygen in the blood^30^. Second, elevated body temperature during exercise stimulates oxygen consumption in the mitochondria and consumes additional oxygen, because the respiratory muscle and heartbeats remain at high activity levels^31,32^. Finally, the secretion of adrenaline and thyroid hormones increases, which stimulates the additional oxygen consumption of the mitochondria until these hormones are treated in the circulating blood^29^. These results are consistent with previous studies showing increased oxygen consumption during recovery after high intensity interval exercise because of increased oxidative metabolism to supplement energy expenditure after exercise^24,28,29^.

Although we did not study mechanisms in this study, previous studies and literature have identified potential reasons for increase in EPOC after IEx and AEx. High-intensity exercise stimulates an increase in HR, ventilation, body temperature, and sympathetic nerves as compared to low-intensity exercises and maintains that for a long time^33^. IEx uses the glycolytic system in the body in the high-intensity section than in moderate-intensity aerobic exercise, resulting in higher rates of glycogen decomposition in the muscle, which increases oxygen consumption with fatty acid metabolism for more active glycogen homeostasis in the body after exercise^34^. Besides, Sloth et al.^35^ reported that an increase in oxidative capacity in skeletal muscle affects fat metabolism during post-exercise stability, stressing the importance of EPOC due to improvements in glucose homeostasis and insulin sensitivity. Thus, as reported in several prior studies, it is suggested that the IEx will be more effective in preventing and improving obesity by not only promoting oxidative capacity and local metabolism, but also by increasing oxygen consumption in the post-exercise recovery period.

In order to reduce body fat, an overall increase in energy consumption and prolonged exercise of >30 minutes is recommended^9^. However, the downside is that these aerobic exercises are time-consuming and tedious, which leads to lower prevalence of exercise^36^. Indeed, recent recommendations regarding the physical activity for public health have highlighted that the positive effects of moderate‐intensity endurance exercise can be accrued by repeating bouts of exercise, each lasting >10 minutes. Murphy et al.^19^ reported that total duration of exercise similarly benefits overall health, blood pressure, and TG reactions, as do repeated and continuous exercises of the same duration. Furthermore, Goto et al.^37^ reported that dividing the accumulated exercise of total 30 minutes into 10-minute durations results in a similar 30-minute exercise. 

The EPOC related variables were significantly higher in AEx as compared to CEx. Darling et al.^38^ also reported an increase in post-exercise energy expenditure of AEx as compared to that of CEx. AEx is the amount of daily exercise performed over a day in short bouts, and has effects similar to that of IEx, because the intermittent resting has a positive effect on the sustained increase in energy consumption, which is attributed to the exercise period, and contributes to fat oxidation^39^. This increase is probably related to sympathetic activation^40^. Additionally, the CEx and AEx results of participants with metabolic syndrome showed similar levels of improvement in blood pressure, blood sugar, and blood cholesterol, indicating the similar health training effects^19,37^. In particular, people with obesity should perform repeated exercises for a short period to maintain health. Comparing this to >30 minutes of exercise at a time, it is generally seen that the same health benefits are achieved^19^. However, since AEx is more effective than CEx for fat metabolism, it can be actively recommended for those who want to control weight.

In this study, energy expenditure during recovery was higher after IEx and AEx as compared to CEx. This is an important result in terms of body composition and metabolism of NWO women. Based on the results of this study, it is necessary to consider the condition and energy consumption of the participants. Thus, IEx is the recommended, effective, and practical approach for health and optimization, whereas AEx, which divides total exercise into shorter bouts of exercises per day instead of just 1 exercise, is proposed to be effective in NWO women. Thus, it seems that weight-related problems can be successfully solved through long-term and CEx prescription for NWO women.

In conclusion, IEx and AEx showed empowering EPOC effects. Our study confirmed that when homogenizing the energy expenditure between CEx, IEx, and AEx on an ergometer, EPOC was higher following IEx and AEx as compared to CEx in NWO women aged >20 years. Therefore, we suggest that IEx and AEx could be more effective strategies than CEx in improving body fat and increasing energy expenditure. Although this study had limitations because the underlying mechanisms of improved energy expenditure in response to IEx and AEx were not elucidated, the present findings provide an applied perspective of the benefits of increased energy expenditure with IEx and AEx on the health of NWO women.
